# ‘Weaving of perspectives’: lessons from co-production within an international mental-health science collaboration

**DOI:** 10.1136/bmjment-2026-302940

**Published:** 2026-09-12

**Authors:** James Downs, Adeola Agunbiade, Mariana Bolivar, Maya Kaiser, Claire Friedrich, Tate Kambeu, Emma Kolaru, Gin S Malhi, Clara Martins De Barros, Richardson Mojica, Daniel Mumo, Tawanda Murepa, Kelvin Opiepie, Jennifer Potts, Martin Walker, Emily Wheeler, Lawrence A White, Simonne Wright

**Affiliations:** 1 Independent Researcher, Cardiff, UK; 2 Independent Researcher, Abuja, Nigeria; 3 Transforming Mental Health, London, UK; 4 Independent Researcher, Paris, France; 5 Psychiatry, University of Oxford, Oxford, UK; 6 Independent Researcher, Harare, Zimbabwe; 7 Sydney Medical School, University of Sydney, Sydney, New South Wales, Australia; 8 Independent Researcher, Epsom, UK; 9 Communication, St Dominic College of Asia, Bacoor, Cavite, Philippines; 10 Public Health, Kenya Medical Research Institute, Nairobi, Nairobi, Kenya; 11 University of Botswana, Gaborone, Botswana; 12 Department of Psychiatry, University of Oxford, Oxford, UK; 13 NIHR Oxford Health Biomedical Research Centre, Oxford, UK; 14 Cochrane, London, UK; 15 Department of Health and Social Services, Government of Northwest Territories, Yellowknife, Northwest Territories, Canada; 16 Department of Psychiatry, Stellenbosch University, Cape Town, South Africa; 17 Department of Clinical, Neuro- and Developmental Psychology, Vrije Universiteit Amsterdam, Amsterdam, Netherlands

## Abstract

Co-production is increasingly recognised as central to improving the quality, relevance and ethical integrity of mental health science, yet evidence is still needed on how lived experience involvement can be meaningfully embedded and sustained in complex research settings. Global Alliance for Living Evidence on Anxiety, Depression and Psychosis (GALENOS) is a Wellcome-funded collaboration conducting living systematic reviews and evidence triangulation, with people with lived experience contributing as Experiential Advisors and through a Global Lived Experience Advisory Board. This perspective uses GALENOS as a case example to reflect on what meaningful co-production practically requires especially in the context of large-scale, technical and international collaborations. It draws on a collective reflection designed and led by people with lived experience, developed through workshops and written contributions involving lived experience contributors, researchers and project-support staff. Discussions were organised around lived experience involvement, capacity development, diversity and representation and influence in strategic decision-making. Across these areas, contributors identified that experiential knowledge needs to become consequential, participation must be supported through care and flexibility, diversity must shape interpretation rather than representation alone and influence must be embedded in governance and decision-making. GALENOS shows that co-production is not a fixed method but an evolving relational practice requiring practical infrastructures for accessibility, accountability, continuity and ethical care.

## Background

 Co-production is now widely recognised as central to improving the quality, relevance and impact of mental health science.^1–3^ The inclusion of people with lived experience has therefore become an established expectation across funding, academic and clinical domains.^4 5^ Yet translating this principle into everyday research practices remains difficult, particularly within large-scale scientific collaborations where professional, clinical, methodological and experiential forms of knowledge may be differently valued. Epistemic injustice remains a persistent concern, understood as the unfair treatment of people with lived experience in their capacity to know and create knowledge.^6 7^ Further work is needed to understand how research can better navigate these forms of knowledge,^7 8^ support diverse contributors equitably^9 10^ and avoid participatory practices that reproduce existing hierarchies under the guise of inclusion.^11 12^

The Global Alliance for Living Evidence on aNxiety, depressiOn and pSychosis (GALENOS) provides a distinctive example for examining these tensions within contemporary mental health science. GALENOS is a Wellcome-funded international collaboration that conducts living systematic reviews (LSRs) on pressing subjects in mental health (https://www.galenos.org.uk/). The GALENOS Approach to Triangulating Evidence (GATE) has been developed as a novel method for bringing together human, animal and mechanistic research alongside clinical, methodological and experiential perspectives. Structured triangulation meetings examine convergence, divergence, gaps in knowledge and implications for real-world settings.^13^

Co-production is embedded throughout. People with lived experience contribute as Experiential Advisors and through the GALENOS Lived Experience Advisory Board (GLEAB), alongside researchers, clinicians and methodologists. Each LSR is co-designed, with experiential input informing research questions and subsequent review stages. GATE brings together multiple sources of experiential, academic, clinical and methodological expertise to address heterogeneity and strengthen interpretive depth,^13–15^ and lived experience contributors are supported through preparatory materials, training and individually tailored support. Triangulation meetings are co-chaired by methodological and lived experience leads, supporting parity of voice.^13^

This Perspective article was developed through a structured process of collective reflection rather than formal qualitative research, designed and led by people with lived experience. As such, it did not require ethical approval. GLEAB members initially identified four areas they considered particularly important for reflection: navigating lived experience involvement, capacity development, diversity and representation and influence in strategic decision-making. These topics served as prompts for three online workshops open to lived experience contributors, researchers and project-support staff, who were invited to respond from their individual perspectives and to discuss where experiences, interpretations and priorities differed. Written reflections could also be submitted alongside or instead of workshop participation. The total author group comprised 10 lived experience contributors and eight researchers or members of the project-support team, with further details of roles, affiliations and geographic distribution provided in online supplemental appendix 1.

**Figure 1:**
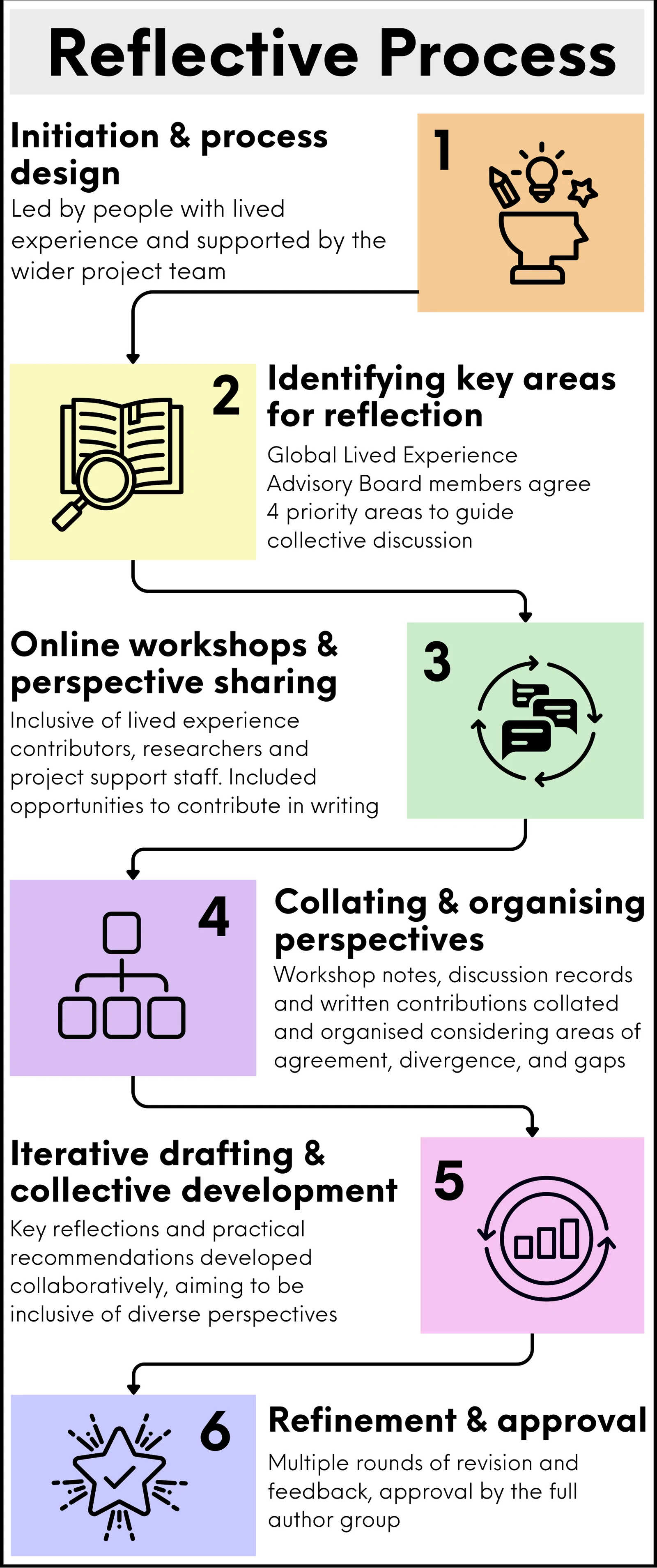
A structured process of shared reflection.

Workshop notes and written reflections were collated to provide a basis for examining different experiences and perspectives across the collaboration but were not subjected to formal qualitative analysis. The key reflections and practical recommendations presented in this paper were developed iteratively through rounds of drafting, circulation and discussion, with all authors contributing to revising and approving the final account. Illustrative quotations were selected to convey the range and substance of perspectives shared by contributors. Figure 1 summarises this structured reflective process.

## Key reflections

### Creating conditions for confident and sustainable participation

Co-production was collectively understood as a fundamentally relational and mutual process. This means that preparation for co-production needs to extend beyond training people with lived experience to participate in existing research practices. While introductory sessions on systematic reviews and evidence synthesis for people with lived experience were deemed necessary, lived experience contributors also emphasised the importance of flexible participation, visible feedback and reciprocal learning throughout. For example, one noted, ‘Sharing the slides and materials after the workshop, and sometimes allowing feedback later to give us additional time for reflection, was very helpful as well’. Practices such as these helped accommodate diverse pacing, communication and processing needs, aligning with participatory approaches that emphasise adaptation and reciprocity as ethical conditions of collaboration.^5 8 16^

The emotional dimensions of co-production were also seen as centrally important. One contributor observed that ‘those with lived experience of mental illness will have good and bad days, be it mood, brain fog, talking too much or going too far the other way and not feeling anything’ while another noted that ‘contributing your lived experience for research can take an emotional toll as you may have to relive quite painful experiences’. These accounts show how the incorporation of experiential knowledge can be both valuable and potentially exposing,^17 18^ and how practices that enhance flexibility and allow for emotional decompression can create more supportive conditions. The broader lesson that emerged is that relational care and methodological quality are not separable, meaning co-production requires relationships and infrastructures that allow people to contribute with confidence, dignity and safety.^5 16 17^

### Making experiential knowledge consequential

A recurring reflection was that meaningful co-production depends not only on inclusion, but on creating conditions in which experiential knowledge can enter technical scientific discussions on its own terms. Technical language initially created uncertainty for some, with one contributor describing ‘feeling unsure how my lived experience fit alongside technical research-intensive discussions. At times, the language and methods felt distant from my world. This was addressed by creating space for open dialogue, breaking down complex terms and encouraging me to share insights in my own words. Over time, this not only made me feel included but also showed that my perspective brought value that the research truly needed’. This reflection demonstrates how adaptive practices can facilitate inclusion and create shared interpretive ground across different ways of knowing.^6–8 16^

However, contributors also highlighted that it was sometimes unclear whether, and how, lived experience input had been taken forward within the project. One noted, ‘I FEEL that it was but I don’t know for sure that it was. It was mentioned in an email but I don’t know if it was common courtesy’. This distinction - between being heard and knowing one has genuinely influenced the work - is central to considerations of ‘meaningfulness’ in co-production.^4 11^ Another contributor emphasised the role of equitable dialogue in enhancing the quality of research, saying: ‘these conversations and weaving of perspectives is where the learning and insights arise…and we lose this rich data when we do not engage in any conversation’. Together, these reflections suggest that meaningful co-production depends on clear routes through which lived experience can enter scientific discussion on its own terms, acting to shape interpretation, prioritisation and decision-making rather than remaining at the level of consultation.^4 12 19^

### Seeking diversity as an active and epistemic practice

Diversity was not understood as a static characteristic of the group but as an active process requiring care, flexibility and intention. As one contributor put it, ‘It’s not enough to just accept diversity. Diversity has to be sought out’. Contributors described moments in which perspectives rooted in under-represented contexts reshaped the work, with one reflecting that ‘a couple of research questions were added to a certain project from my contribution. I brought in a resource-scarce aspect’. Here, the contributor was referring to the inclusion of questions informed by settings in which mental health services, research infrastructure and other resources may be limited. Such examples show how contributors from different disciplines, cultures, geographies and lived experiences can expose gaps in dominant framings and reorient research priorities, generating both creativity and complexity.^9 10^

At the same time, there was agreement that diversity operates most powerfully at the level of interpretation rather than representation alone. One contributor observed that ‘these different viewpoints can conflict and layer in interesting ways’, highlighting the productive but sometimes demanding work of negotiating meaning across perspectives. Whilst structured triangulation meetings created spaces where experiential, clinical and methodological perspectives could interact, contributors also identified absent perspectives including those from Indigenous communities, rural African communities and low-income or otherwise marginalised groups. This points to the need for research to take a more active role in diversifying who is involved and enabling difference to shape the questioning and interpretation of evidence.^9 10 19^

### Embedding meaningful influence in strategic decision-making

Contributors described changes resulting from lived experience input, with one saying ‘it has been meaningful to have a say in how the project has evolved and to see tangible changes in the way we do things as a result of my input into decisions’. Such changes included introducing a lived experience co-chair for triangulation meetings, providing simplified materials and matching Experiential Advisors to projects aligned with their expertise.^13^ This demonstrates how co-production can reshape the conditions of research as well as its content and focus.

At the same time, contributors emphasised that influence depends on factors including clarity, continuity and feedback. One described how influence ‘could be improved by involving people with lived experience early on in decision-making, giving more space for more input throughout the project and ensuring feedback loops so we can see how our contributions directly shape outcomes’. These reflections indicate that co-production is strongest when embedded at the level of strategic decision-making and leadership and when contributors can see how their input travels throughout the research collaboration.^4 20^

## Sustaining co-production in mental health science

Lessons from the GALENOS project reflect a broader challenge facing contemporary mental health science: the need to move from establishing the presence of co-production to ensuring that lived experience meaningfully shapes the priorities, practices and performance of research.^4 6 7^ Lived experience input was seen as most meaningful when it tangibly changed the framing, interpretation and prioritisation of evidence, rather than being incorporated symbolically. This supports wider critiques of participatory research which argue that inclusion alone does not necessarily disrupt established hierarchies of credibility and authority.^11 12 19^

Co-production is best understood as an ongoing and relational practice, not a stable methodological achievement. Across GALENOS, accessible materials, asynchronous participation, lived experience co-chairing, continual feedback loops and individually tailored support were all part of the evolving infrastructure of co-production required to support more equitable and ethical participation throughout.

Table 1 highlights some of the practical implications of the reflections presented in this paper. They represent areas of broad agreement across the group rather than the results of a formal consensus exercise; where contributors emphasised different priorities, these were retained within the breadth of the recommendations.

**Table 1:** Recommended conditions for ethical and sustainable lived experience collaboration

Foundational prerequisites	
Well-being and ethical care	Embed emotional support as an ethical requirement of collaboration, with regular check-ins and clear options to pause or step back. Safeguarding well-being is integral to sustaining participation and preventing harm.
Inclusion and accessibility	Broaden participation among under-represented groups, including Indigenous communities, low-income populations, and contributors from the Global South. Ensure engagement methods accommodate differences in access, language, time and cognitive or communication needs.
Reflective training for researchers	Extend training beyond orientation for experiential advisors to include structured reflection for researchers on power, language and emotional labour in collaboration.
Relational and operational essentials	
Two-way feedback and visible impact	Implement systematic feedback loops that show how experiential input has informed decisions, supporting accountability and reducing uncertainty.
Long-term support and learning	Monitor contributors’ skills, confidence and well-being over time, with sustained mentorship and peer learning embedded within project structures.
Consistency with flexibility	Establish clear standards for roles, remuneration and communication while retaining flexibility across contexts. Consistency should ensure fairness not uniformity.
Refining collaborative practice	
Representational frameworks for lived experience	Develop structured guidance to clarify when contributors speak from personal experience and when they represent collective perspectives, supporting transparency and interpretive coherence in triangulation.
Contributing to learning and capacity building	Share insights with the research community (including what worked and how participation efforts can be improved) as part of broader efforts to build capacity in the lived experience workforce and support field-wide competence in coproduction methodologies.

GALENOS also highlights some key challenges that remain unresolved. International collaboration can widen the range of perspectives informing evidence generation, but it does not automatically overcome the dominance of English-speaking and Global North contexts.^9 10^ Similarly, involving people with lived experience does not in itself ensure influence over governance, interpretation or implementation. Future co-produced research should therefore shift attention towards increasing diversity, transparency and evaluation in order to ensure experiential knowledge becomes consequential within research systems.^4 20^

## Supplementary material

10.1136/bmjment-2026-302940 online supplemental file 1
