# Supplementary material for: ‘Weaving of perspectives’: lessons from co-production within an international mental-health science collaboration
Source: BMJ Ment Health. 2026 Sep 12;29(1):e302940. doi: 10.1136/bmjment-2026-302940 (PMC13583850; doi:10.1136/bmjment-2026-302940)
Supplement: Supplementary file 1 [file bmjment-29-1-s001.docx]

**Appendix 1.** Roles, affiliations and geographic distribution of the author group

| **Name** | **Role within the project** | **Occupational category** | **Organisation** | **Country** |
| --- | --- | --- | --- | --- |
| Adeola Agunbiade | GLEAB member | Lived experience advisory | Independent | Nigeria |
| Mariana Bolivar | MQ Research Lead | Project team | MQ, Mental Health Research, London | Columbia |
| James Downs | Experiential Advisor | Lived experience advisory | Independent | UK |
| Claire Friedrich | GALENOS Researcher | Project team | University of Oxford | UK |
| Maya Kaiser | Experiential Advisor | Lived experience advisory | Independent | France |
| Tate Kambeu | Chair of the GLEAB | Lived experience advisory | Independent | Zimbabwe |
| Emma Kolaru | MQ Project Manager | Project team | MQ, Mental Health Research, London | UK |
| Gin Malhi | GALENOS Co-Chair of Engagement and Impact | Project team | University of Sydney | Australia |
| Clara Martins de Barros | Experiential Advisor | Lived experience advisory | Independent | UK |
| Richardson Mojica | Vice Chair of the GLEAB | Lived experience advisory | #MentalHealthPH / St. Dominic College of Asia | Philippines |
| Daniel Mumo | Experiential Advisor | Lived experience advisory | Kenya Medical Research Institute (KEMRI) | Kenya |
| Tawanda Murepa | Experiential Advisor | Lived experience advisory | Independent | Zimbabwe |
| Kelvin Opiepie | GLEAB member | Lived experience advisory | Independent | Nigeria |
| Jennifer Potts | GALENOS Project Manager | Project team | University of Oxford | UK |
| Martin Walker | Patient and Public Involvement Officer | Project team | Cochrane | UK |
| Emily Wheeler | MQ Director of Research Partnerships and Development | Project team | MQ, Mental Health Research, London | UK |
| Lawrence A. White | GLEAB member | Lived experience advisory | Independent | Canada |
| Simonne Wright | GALENOS Fellow | Project team | Stellenbosch University | South Africa |
